# Patient attitudes towards medical students at Damascus University teaching hospitals

**DOI:** 10.1186/1472-6920-12-13

**Published:** 2012-03-22

**Authors:** Rima M Sayed-Hassan, Hyam N Bashour, Abir Y Koudsi

**Affiliations:** 1 Department of Internal Medicine, Faculty of Medicine, Damascus University, Damascus, Syria; 2 Department of Family & Community Medicine, Faculty of Medicine, and Centre for Medical Education Development, Damascus University, Damascus, Syria; 3 Department of Family & Community Medicine, Faculty of Medicine, Damascus University, Damascus, Syria

## Abstract

**Background:**

The cooperation of patients and their consent to involve medical students in their care is vital to clinical education, but large numbers of students and lack of experience as well as loss of privacy may evoke negative attitudes of patients, which may sometimes adversely affect the clinical teaching environment. This study aimed to explore the attitudes of patients towards medical students at Damascus University hospitals, and to explore the determinants of those attitudes thus discussing possible implications applicable to clinical teaching.

**Methods:**

This cross-sectional study was conducted at three teaching hospitals affiliated to the Faculty of Medicine at Damascus University. Four hundred patients were interviewed between March and April 2011 by a trained sociologist using a structured questionnaire.

**Results:**

Of the patients interviewed, 67.8% approved the presence of medical students during the medical consultation and 58.2% of them felt comfortable with the presence of students, especially among patients with better socio-economic characteristics. 81.5% of the patients agreed to be examined by students in the presence of the supervisor, while 40.2% gave agreement even in the absence of the supervisor. Privacy was the most important factor in the patients' reticence towards examination by the students, whilst the relative safety and comfort if a supervisor was available determined patients' agreement.

**Conclusions:**

The study concluded overall positive attitudes to the medical students' involvement in medical education. However, it is essential that students and clinical supervisors understand and adhere to professional and ethical conduct when involving patients in medical education.

## Background

Clinical teaching is central to medical student education [1,2]. Contact with patients remains a vital component in the education of medical students. Encounters with patients can promote contextual and clinical learning, improve communication and professional skills and initiate the development of a future doctor-patient relationship [3]. Patients' willingness to cooperate and contribute to the education and training of medical students provide better teaching opportunities for students at most levels of patient care [3,4].

Modern healthcare consumers are active participants in choosing their care, leading to difficulty in training students if patients decline their involvement. With increased focus on patients' rights and informed consent, patients can and should now choose whether to have medical students present during their consultations. However, conflict can arise between the educational requirements of medical students and the needs of the patients [5,6].

One of the earliest studies conducted at the University of Manchester in 1974 showed that 40% of patients do not like to discuss family problems, sexual or anxiety disorders in the presence of medical students [7]. A number of later studies in a variety of settings and specialties have shown that patients are willing to participate in the education of medical students, and that satisfaction is generally positive after participating in undergraduate medical education [8-11], citing reasons such as a desire to contribute to medical education, the extra time presenting physicians may spend with the patient, and being able to increase their own knowledge about their medical problem [9-14]. Studies frequently mentioned another motive which was altruism meaning that patients felt good about contributing to the student's education [8,11,12,15].

While feedback from patients is largely and generally positive regarding medical student participation as being shown in the comprehensive review of Mol and colleagues [16] and this is regardless of the nature of specialty with little intrinsic differences between specialties [17,18], Simons and co-authors showed that 55.8% of the patients had no preference regarding medical student participation, while a third preferred to see the attending physician alone [19]. Generally, studies showed that there is little reluctance to students' presence for emotional or intimate problems; expectedly, in Obstetrics and Gynecology as well as in Genito-Urinary clinics approval of students' involvement is reported to be lower [20-23].

In the Arab World, similar studies were conducted. Despite specificities of some social and cultural aspects in the Arab World, broad acceptance by patients to medical students was noted [24-26].

In the absence of similar studies in Syria and with regard to the national efforts for quality improvement in both medical education as well as health care standards, it was necessary to explore the opinion of patients and what they felt and the degree of their comfort upon examination by an unspecified number of students. Medical education in Syria is characterized by being very traditional, with large number of students and hospital-based education; in which clinical teaching takes place in the fourth to sixth years of medical training. Serious problems of clinical teaching at Damascus University Faculty of Medicine were documented in a recent study [27].

This study was designed to identify the attitudes of Syrian patients seen at three teaching hospitals affiliated to Damascus University towards medical students' presence and their comfort level during physical examination, their preferences regarding medical student involvement; and to identify factors that determine those attitudes, mainly the type of the hospital and patients' characteristics.

## Methods

This is a cross-sectional study that relied on conducting face-to-face interviews with a random sample of patients admitted to the three main teaching hospitals, two general and one maternity, affiliated to the, Damascus University Faculty of Medicine. These three hospitals contribute to 68.5% of total number of beds in all teaching hospitals of Damascus University. This study was carried out during the period from beginning of March to end of April 2011.

The study recruited a sample proportional to the number of beds in each hospital allowing for all specialized units. Thus the study sample included 400 patients distributed into 100 patients in Al Assad University Hospital, 175 in Al Mouassat University Hospital and 125 in the Maternity University Hospital. In each hospital, a systematic random sample using the admission office list as to represent all patients in large units (departments) was selected. Within each department, another systematic random selection of patients within small units (divisions) was done.

The questionnaire was designed specifically for the purpose of the study having consulted the medical literature of similar studies [8-11,24,28,29]. The questionnaire was piloted on a sample of 15 patients to ensure face validity and clarity. Minor changes were made; namely rewording two questions. Those interviews were excluded from the analysis. The questionnaire contained 46 items, including demographic and socio-economic data and patients' attitudes toward medical students and their preferences when involving medical students in the clinical examination.

Face-to-face interviews using the questionnaire were conducted by one trained sociologist to reduce potential bias in the event of data were collected by the medical team. Interviews took place at a calm corner of wards.

The Institutional Review Board approved the study proposal and ethical considerations were respected by taking the informed consent from all patients who were interviewed.

Data were entered into a Microsoft Excel spreadsheet and the statistical analysis was conducted using SPSS (Version 17). Statistical analysis was carried out using descriptive and analytical statistics. Simple frequencies and cross tabulation were done. Chi square test was used for proportions. ANOVA test was used to test for statistical significance testing when comparing means. Stratification for the patient's sex and educational level was done when relevant. P value of less than 0.05 was considered statistically significant.

## Results

Four hundred patients were interviewed in this study. None of the patients approached refused to participate. General characteristics of patients recruited in the study sample, according to the hospital they came from, are presented in Table 1. The average age of patients was 40.2 years (SD = 15.2), and 14.7% of them had high school or university level education. Most patients came from Damascus or its surroundings (62.3%), while the rest came from other provinces all over the country. The table clearly shows the disparity in the characteristics of patients between the three hospitals, where patients seen at Al Assad University Hospital had better socio-economic characteristics (such as education, income) as compared to other hospitals, and the differences were statistically significant.

**Table 1:** General characteristics of patients in the study sample by hospital•

Item	Al Assad University Hospital (100) No (%)	Maternity University Hospital (125) No (%)	Al Mouassat University Hospital (175) No (%)	All patients (400) No (%)
Sex*				

Male	61 (61)	-	75 (42.9)	136 (34)

Female	39 (39)	125 (100)	100 (57.1)	264 (66)

Marital Status *				

Single	14 (14.1)	5 (4.1)	43 (24.7)	62 (15.7)

Married	75 (75.8)	115 (93.5)	117 (67.2)	307 (77.5)

Divorced/Widow	10 (10.1)	3 (2.4)	14 (8)	27 (6.8)

Educational Level *				

Illiterate/reads & Writes	31 (31.3)	29 (24)	62 (35.6)	122 (31)

Primary/Preparatory	36 (36.4)	78 (64.5)	100 (57.5)	214 (54.3)

Secondary/University	32 (32.3)	14 (11.6)	12 (6.9)	58 (14.7)

Age				

Mean (SD)	49.6 (15.5)	31.9 (11.7)	40.7 (14.3)	40.2 (15.4)

Number of family members				

Mean (SD)	5.4 (2.4)	5.9 (4.2)	7.4 (4.4)	6.4 (4)

Average monthly income (SYP)				

Median	20000	13700	15000	15000

* P < 0.001

**•**Total might be less than 400 due to missing values

**Figure 1:**
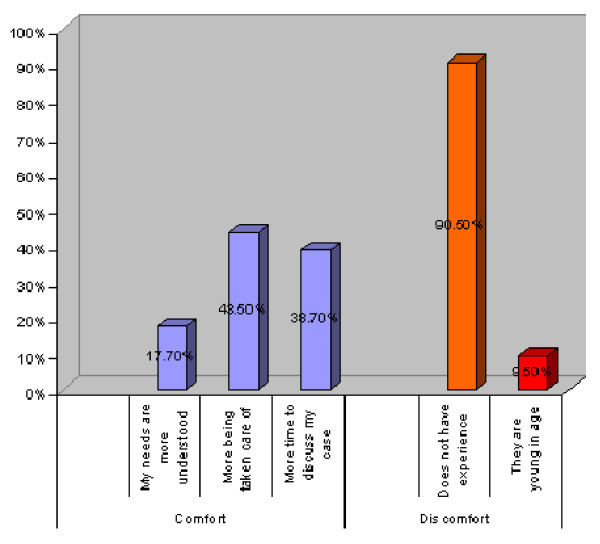
**Reasons reported by patients for their comfort or discomfort with medical students' involvement**.

Table 2 summarizes the patients' attitudes towards the education of medical students which seemed generally positive, with a statistically significant difference between the hospitals. Of the patients interviewed, 67.8% approved the presence of medical students during the medical consultation, and 58.2% expressed comfort with the presence of medical students, and this percentage was higher in the hospital where socially and financially better off patients were seen (70.1%). Figure 1 shows the reasons for comfort expressed by the patients where the desire to get better attention if their cases were lengthily discussed was reported to be the most important reason for the comfort and satisfaction of patients while the lack of students' experience (90.5% of all causes) was the main factor for discomfort with the presence of students.

**Table 2:** Patients' attitudes towards medical students learning by hospital•

Item	Al Assad University Hospital (100) No (%)	Maternity University Hospital (125) No (%)	Al Mouassat University Hospital (175) No (%)	All patients (400) No (%)
Do you approve the existence of medical students during clinical encounter? *				

Yes	82 (82.8)	70 (56)	118 (67.8)	270 (67.8)

No	4 (4)	32 (25.6)	23 (13.2)	59 (14.8)

Does not matter	13 (13.1)	23 (18.4)	33 (19)	69 (17.3)

Do you feel comfortable in the existence of medical students? *				

Yes	68 (70.1)	64 (51.2)	99 (56.6)	231 (58.2)

No	5 (5.2)	11 (8.8)	25 (14.3)	41 (10.3)

Does not matter	24 (24.7)	50 (40.0)	51 (29.1)	125 (31.5)

Do you approve being examined by medical students under supervision? *				

Yes	96 (96)	91 (72.8)	139 (79.4)	326 (81.5)

No	3 (3)	30 (24)	29 (16.6)	62 (15.5)

Does not matter	1 (1)	4 (3.2)	7 (4)	12 (3)

Do you approve being examined by medical students in the absence of supervision? *				

Yes	47 (47)	27 (21.6)	86 (49.7)	160 (40.2)

No	44 (44)	94 (75.2)	79 (45.7)	217 (54.5)

Does not matter	9 (9)	4 (3.2)	8 (4.6)	21 (5.3)

Do you approve having your clinical case discussed in front of you by clinical staff and medical students?				

Yes	89 (90.8)	113 (91.9)	145 (83.3)	347 (87.8)

No	8 (8.2)	9 (7.3)	27 (15.5)	44 (11.1)

Does not matter	1 (1)	1 (0.8)	2 (1.1)	4 (1)

Does the part of your body to be examined affects your approval of involving a medical student in the clinical exam? ‡				

Yes	30 (30.6)	104 (83.9)	46 (27.1)	180 (45.9)

No	59 (60.2)	14 (11.3)	92 (54.1)	165 (42.1)

Does not matter	9 (9.2)	6 (4.8)	32 (18.8)	47 (12)

Do you think that you have the right to approve or disapprove the existence of medical students during the clinical encounter? * ‡				

Yes	19 (19.2)	45 (36)	77 (44)	141 (35.5)

No	56 (56.6)	50 (40)	62 (35.4)	168 (42.1)

Does not know	24 (24.2)	30 (24)	36 (20.6)	90 (22.6)

* P < 0.001

‡ P < 0.05 after stratifying for sex of the patient

**•**Total might be less than 400 due to missing values

**Figure 2:**
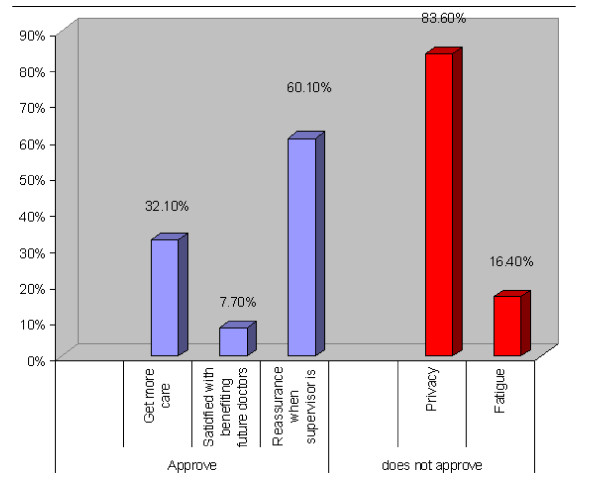
**Reasons reported by patients for approving or disapproving clinical exams by medical students under supervision**.

Patients' attitudes towards medical students are presented in Table 2. Of all patients, 81.5% approved to be examined by a medical student in the presence of a supervisor, while 40.2% approved the examination in the absence of a supervisor. Figure 2 illustrates the reasons, where privacy seemed to be the most important factor in patients rejecting the examination by students (83.6% of reasons for rejection). Feeling of safety and confidence if a doctor is in charge was the most important factor in patients approving the examination by the student (60.1% of the reasons for acceptance).

As for results with regard to the part of the body being examined (Table 2), women in the Maternity University Hospital refused the examination, especially when parts examined are obviously sensitive for women. Of all women interviewed at the Maternity University Hospital, 83.9 referred to the part from the body examined as being determining their acceptance versus 27.1% of patients seen in Al Mouassat Hospital with internal and surgical cases are managed. It was striking to note that over one-third (35.3%) of the study sample were aware of their right to refuse or accept the presence of medical students during the clinical encounter, and this varied largely between the hospitals to be significantly lower in the hospital receiving the better educated patients (19.2% in Al-Assad Teaching Hospital, compared to 44% in Al Mouassat Hospital).

Patients' preferences regarding the teaching of medical students are shown in Table 3. Patients preferred a smaller number of students during the clinical round (72% of patients preferred the presence of a maximum of 9 medical students) and would prefer to be examined by one or two students only (59.3%). The sex of the student was also an important determinant where the majority of women seen at the Maternity Hospital would prefer that the clinical examination be done by a female medical student (59.7% in Maternity hospital vs. 14.6% in Al-Assad University Hospital).

**Table 3:** Patients' preferences with regard to medical students learning by hospital

Item	All patients (400) No (%)	Al Mouassat University Hospital (175) No (%)	Maternity University Hospital (125) No (%)	Al Assad University Hospital (100) No (%)
Do you prefer to be examined by? * ‡				

Medical student with same sex as yours	124 (31.6)	36 (20.8)	74 (59.7)	14 (14.6)

No difference	236 (60.1)	121 (69.9)	36 (29)	79 (82.3)

I do not agree in all cases	33 (8.4)	16 (9.2)	14 (11.3)	3 (3.1)

What is your preferred number of students to exist during clinical consultation? * ‡				

Nil	4 (1)	3 (1.7)	1 (0.8)	- (-)

1-3	58 (14.6)	36 (20.8)	16 (12.8)	6 (6.1)

4-8	224 (56.4)	94 (54.3)	82 (65.6)	48 (48.5)

9 or more	111 (28)	40 (23.1)	26 (20.8)	45 (45.5)

What is your preferred number of students to examine you during clinical consultation? * ‡				

Nil	107 (26.8)	71 (40.6)	29 (23.2)	7 (7)

1-2	237 (59.3)	80 (45.7)	92 (73.6)	65 (65)

3-5	43 (10.8)	18 (10.3)	3 (2.4)	22 (22)

6 or more	13 (3.3)	6 (3.4)	1 (0.8)	6 (6)

Do you prefer to have the clinical examination by the medical students? * ‡				

At the same time	138 (35.8)	56 (32.4)	47 (40.9)	35 (36.1)

One visit by a group	23 (6)	8 (4.6)	6 (7.8)	6 (6.2)

Frequent visits by students	73 (19)	20 (11.6)	24 (20.9)	29 (29.9)

Does not matter	151 (39.2)	89 (51.4)	35 (30.4)	27 (27.8)

* P < 0.001

‡ P < 0.05 after stratifying for sex of the patient

**•**Total might be less than 400 due to missing values

## Discussion

This study contributed to the understanding of Syrian patients' attitudes towards the involvement of medical students in clinical teaching, as reported by those seen at three main teaching hospitals at Damascus University. Overall, the degree of acceptance of medical students was high, similarly to what was reported in many other studies from developed countries [8-16] and the Arab World [24-26]. It is worth mentioning that this trend was dominant in the three teaching hospitals studied despite the different socio-economic characteristics of their patients. It is worthy to note that Al Assad Hospital received patients referred from governmental and some private establishments while the service provided at Al Mouassat University Hospital and the Maternity Hospital is less organized, and in the latter two hospitals the services are completely free of charge.

This study also revealed reasons for which patients felt comfortable with the presence of medical students. Those reasons were indeed similar to those reported in the literature including the desire to contribute to medical education, the extra time spent with the patient, and the opportunity to learn more about their medical problem [9-14,24,25]. Although the need to get patients' consent is a must [3], our study unfortunately revealed that patient consent is simply absent as more than two-thirds of patients were indeed unaware of their rights to refuse or accept the active involvement of the medical students. O'Flynn and colleagues reported that 28% of patient thought that they did not have a choice about student presence and participation [13], while Abdulghani and colleagues reported that 45.1% of patients believed that they had not the right to refuse medical students [24]. The study of Chipp and colleagues showed that 89% of patients admitted that they would expect to have their permission sought before seeing a student [30]. This issue is very critical, as the need to humanize the medical education is very evident, especially in settings such as ours where patients are the main educational "tools," since clinical skills lab was not introduced until quite recently. Inpatients at hospitals are mainly used for clinical teaching while outpatients are very rarely involved.

It was of interest to find that feeling of safety and comfort is indeed correlated to the presence of a supervisor. This is largely due to the lack of awareness of the extent of students' involvement. This finding is consistent with other studies [22-25]. On the other hand, privacy was the main reason behind patients discomfort with Students' involvement.

We analyzed our results as to show the differences between the three hospitals when again findings on women seen at the Maternity University hospital was in agreement with other studies [20,22], where all women expressed a preference for students of their same sex and refused male students. This is a pattern that one would expect more in largely Muslim country such as Syria [26]. Of great interest in this work is that differences in attitudes were not only related to the sex of the patients but was also related to their educational level and socio-economic characteristics, which were evident even after stratifying for patient sex. This is contrary to the study of Shah-Khan and colleagues in Chicago that found no relationship between the economic level or degree of educational attainment and the degree of satisfaction of patients [31].

Our study also reported patients' preferences with regard to the scale of medical students' involvement such as the number of students around the bed during the clinical consultation; as well as with regard to the desirable number of students actually examining the patient. Sweeney and colleagues suggested that it is the duty of supervising professor to be aware of the patients' preference taking into accounts the fears and concerns [29]. Our study highlighted the importance of respecting the patients' preferences especially in a context where those are far from the ideal environment of clinical teaching. In our faculty, we face the dilemma of large number of students with small groups as large as 40 students at each point of time.

The cultural and religious background of our patients did not bring any implications on their attitudes towards medical students. This study highlighted similarities with other parts of the world where the humane needs (respect, dignity) of the patients are similar, however one might argue that the implications of over-dependence on real patients in our clinical teaching setting necessitates more notion of the humane need of the patients.

The main strength of our study is that it analyzed findings by type of hospital and allowed for socio-economic factors. Apparently this needs to be considered when taking any future steps into consideration. The main limitation is that potentially patients might have reported their answers without differentiating between undergraduate and postgraduate students. We do not think that seeking care at a free teaching hospital has negatively affected our findings as selection of the hospital was largely driven by external factors.

## Conclusions

Syrian patients showed overall positive attitudes towards the involvement of medical students. They expressed comfort in general but some expressed concerns over the experience of students as well as over breaching confidentiality. Patient's sex as well as their socio-economic background determined their attitudes and preferences. Patients preferred lower number of students to be involved; however, few were aware of their rights. A more ethical and professional environment and practices are urgently needed in our setting. Patients, students as well as clinical teachers need to learn about the ethics of patients' involvement in medical teaching.

## Competing interests

The authors declare that they have no competing interests.

## Authors' contributions

BRSH and HB contributed to the idea of research, research design and supervision of data collection. They have both contributed to drafting the paper. HB and AK performed the statistical analysis. AK also contributed to paper drafting. All Authors read and approved the manuscript.

## Pre-publication history

The pre-publication history for this paper can be accessed here:

http://www.biomedcentral.com/1472-6920/12/13/prepub
